# Supplementary material for: Corporate social responsibility and cross-border M&A: The moderating effect of institutional distance
Source: PLoS One. 2022 Jan 27;17(1):e0262343. doi: 10.1371/journal.pone.0262343 (PMC8794142; doi:10.1371/journal.pone.0262343)
Supplement: Attachment — Submitted filename: Response to Reviewers.docx [file pone.0262343.s001.docx]

**PONE-D-21-18638**

**Response to the Comments of Academic Editor**

**Comments:** Thank you for submitting your manuscript to PLOS ONE. After careful consideration, we feel that it has merit but does not fully meet PLOS ONE’s publication criteria as it currently stands. Therefore, we invite you to submit a revised version of the manuscript that addresses the points raised during the review process.

The quantitative framework should be refined. There are more than necessary robustness checks, along with further interpretations.

**Response:** Thank you very much for your positive remarks on our manuscript. We greatly appreciate for allowing us to revise and improve our manuscript. Following the comments of you, Reviewer 1 and Reviewer 2, we have carefully refined the quantitative framework and clearly clarified the statements. We hope you and the reviewers will find our response and revision work satisfactory.

Please see our responses to Reviewer 1 and Reviewer 2 below.

**Response to the Comments of Reviewer #1**

The subject is interesting, and the paper is overall clear and well written. I hope my comment will enable the authors to improve their work.

**Response:** Thank you very much for your positive remarks on our paper. Following your guide, we have carefully revised the paper. We believe that your comments have improved the quality of this paper significantly.

**Comments:** The study is performed concerning Chinese firms, which is interesting, but the reader has almost no information on the specificities of the Chinese context concerning M&A.

**Response:** Thank you very much for your kind and constructive suggestions. Following your suggestions, we have addressed your concern on the information about the specificities of the Chinese M&A by providing relevant data in our sample years 2010-2016. This point has been clarified in our manuscript (pp.3-4):

Specifically, this study utilizes a sample of Chinese firms as the research context, for two reasons. First, according to the statistics from open sources, the money spent on cross-border M&A transactions by Chinese companies greatly increased, from 34.7 billion dollars in 2010 to 150.6 billion dollars in 2016, and the numbers of transactions climbed from 268 to 738. Furthermore, with the reinforcement of China’s “Go Global” strategy and the Belt and Road initiative, as embodied in the upgrading of corporations, the process of cross-border M&A by Chinese enterprises is growing significantly. For example, Chinese companies investing in these countries conducted no less than 192 M&A transactions in 2016, representing an aggregate value of 19.3 billion dollars, in contrast with 46 transactions and 4.6 billion dollars in 2010. However, the completion rate of cross-border M&A by Chinese enterprises seems below the world average. This outcome may indicate Chinese enterprises may not notice the underlying factors that may lead to failure in their cross-border M&A activities.

Second, China, the largest emerging market with the transition into the market economy, is integrating into the world economy and enhancing the governance quality, thereby attracting more and more academic attention. Considering that there are many common characteristics between China and other emerging markets, this study advances our knowledge of emerging markets. Additionally, it may contribute the stream of literature on M&As through investigating how firms from emerging markets complete cross-border M&A deals, especially considering firms from these regions experience high percentage collapse before completion (Zhou et al., 2016).

**Comments:** The way hypothesis are presented is strange. There are two competing hypothesis at the beginning (H1a and H1b) but afterwards, the authors take a positive relationship for granted for H2 and H3.

**Response:** Thank you very much for your constructive comments. As you suggested, we have refined the hypothesis to more reflect our findings by drawing upon extent studies and focusing on the characteristics of emerging markets. We argue that the positive relationship between CSR of Chinese companies and M&A is more common. Namely, CSR has a significant positive effect on cross-border M&A. That is, the stronger the CSR, the more likely an enterprise will be to complete a cross-border M&A. This part has been clarified in our manuscript (pp.6-7).

Specifically, we suppose that CSR has a positive effect on the completion of M&A for Chinese companies. As characterized by the emerging market, institutions are transitioning to the market economy, their implementation can still be erratic, and their norms are evolving (Bruton et al., 2019). Consequently, firms from emerging markets like China may be likely to suffer the liability of foreignness when conducting cross-border M&A in international markets. Accordingly, as a strategic tactic, CSR enables these firms to enhance their legitimacy in host countries and build trustworthiness with target firms’ stakeholders (Bertrand et al., 2021), thereby overcoming the liability of foreignness (Husted et al., 2016) and facilitating the completion of M&A. Therefore, we suppose that,

Hypothesis 1: CSR has a significant positive effect on cross-border M&A. That is, the stronger the CSR, the more likely an enterprise will be to complete a cross-border M&A.

**Comments:** You are focusing on the CSR on the targeting firm. Why not about the CSR of the target firm?

**Response:** Thank you very much for your constructive comments. As you suggested, we definitely believe that the effect of CSR of the target firm on M&A would be a very interesting topic, so it will be investigated further in our future research. Specifically, current study emphasizes the CSR on the targeting firm rather than the target firm for three reasons.

Firstly, the antecedents on the completion of M&As have been attracting the scholarly attentions. This study aims to explore how firms from emerging market enhance the likelihood of M&A in international markets. To achieve this research objective, we focus on the CSR of the targeting firm and investigate how firms from China overcome the liability of foreignness through increasing their CSR performance. As stated in this study, our findings provide implications for firms from emerging markets.

Secondly, as sated by the prior studies, a variety of reasons from the targeting firm may cause the failure of a deal such as shareholder opposition, financing problems or internal target resistance (Arouri et al., 2019). In other words, the outcome of M&A deals depends on the assessment and opinion of many of the targeting firm’s stakeholders (Arouri et al., 2019). Therefore, it is meaningful and important to discuss the role of targeting firm’s CSR.

Finally, compared to the target firm, it is more important and costly for the targeting firm to complete the cross-border M&A due to upfront financial costs and termination fees as well as losses in terms of firm reputation, credibility, time and diversion of managerial attention (Hawn, 2014).

**Comments:** The stakeholder vs shareholder perspective is sometimes difficult to articulate and I think you should explain more in depth the moderating relationship: why should institutional distance moderate the precise CSR – M&A completion relationship? This is for me not very clear in the paper.

**Response:** Thank you very much for your kind and constructive comments. Following your guide, we further clarified the moderating relationship and strengthen the arguments (pp.7-8).

The main logic is that institutional distance raises the risk and uncertainty of firms’ internationalization and M&A (Kostova et al., 2020), while CSR may reduce the uncertainties (Arouri et al., 2019). Specifically, we develop our arguments from three points (pp.7-8).

Firms looking to invest overseas face a natural disadvantage when they conduct multinational operations as “outsiders”, especially when institutional distance is high. Enterprises entering a host country’s market face mainly the problem of local unfamiliarity caused by information asymmetry, along with discrimination due to the lack of legitimacy and relationship harm as they lack embeddedness. First, the unfamiliarity hazard mainly refers to the fact that overseas enterprises entering a host country’s market are at a disadvantage in understanding the host country’s market situation, legal system, and culture. Obtaining such information often requires a relatively high cost and high engagement in CSR to gain trust and recognition among the host countries’ stakeholders. For example, Kostova and Zaheer (2020) find that when the host and home countries have high institutional distance, CSR is more attractive for foreign firms, and the more positive its CSR activities are, the easier it is for them to correctly understand the host country’s institutional environment, especially informal implicit social norms, cultural practices, religious beliefs, and conventional rules.

Second, the harm caused by the lack of legitimacy refers to the lack of information to judge a foreign firm’s operations in the host country, where locals often rely on stereotypes to judge the behavior of the company. If the foreign firm has higher engagement in positive CSR activities or show successful investment behaviors, then the host country’s stakeholders will believe that the most obvious characteristics are positive and reliable.

Finally, the damage to relationships caused by the lack of embeddedness mainly refers to the fact that a new foreign firm does not effective and close relationships with the host country government, suppliers, consumers, communities, and other stakeholders. Thus, firms establish relations with these parties by engaging in various CSR activities to achieve cross-border M&A. Therefore, we examine the moderating effect of formal and informal institutional distance individually.

**Comments:** I’m not specifically familiar about formal and informal institutions but the moderating effect is strange. The two concepts should be close (and are significantly correlated, see the correlation table). But the results are opposite. My major concern is here. I fear that the model is over identified since you add twice almost the same variable. Even if your VIF are under 10, it has an impact on your coefficients. You should include one variable or the other (formal or informal) but not both.

**Response:** Thank you very much for your insightful comments. We appreciate your kind guidance that helped us significantly improved the quality of our paper. Taking into consideration your concern of the high correlation, we adjust the measurements of formal institutions and informal institutions and then run the regressions of both models containing each interaction term respectively. After adjustment, the coefficient of correlation is lowed to 0.0921 and the empirical results are shown below (pp.18-20).

As reported in Model (3) of Table 6, the interaction term between formal institutional distance and CSR is significantly positive at the 10% confidence level. That is, in the presence of formal institutional distance, companies with high CSR have stronger incentives to conduct M&A activities, supporting Hypothesis 2. Model (4) of Table 6 examines the moderating effect of informal institutional distance, and the interaction term is significantly negative at the 10% confidence level. In other words, the greater informal institutional distance is, the less positive is the impact of CSR on the completion of cross-border M&A. Hence, informal institutional distance negatively moderates the positive impact of CSR on the completion of cross-border M&A, which is contrary to Hypothesis 3.

**Table 6.** **Basic Results and Moderating Effects.**

|  | **(1)** | **(2)** | **(3)** | **(4)** |
| --- | --- | --- | --- | --- |
|  | **Completion** | **Completion** | **Completion** | **Completion** |
| **CSR** |  | 0.354^*^ | 0.392^**^ | 0.354^*^ |
|  |  | (0.191) | (0.195) | (0.195) |
| **Formal Institutional Distance** |  |  | -0.751^***^ |  |
|  |  |  | (0.250) |  |
| **CSR* Formal Institution Distance** |  |  | 0.524^*^ |  |
|  |  |  | (0.310) |  |
| **Informal Institutional Distance** |  |  |  | -0.060 |
|  |  |  |  | (0.179) |
| **CSR* Informal Institution Distance** |  |  |  | -0.390^*^ |
|  |  |  |  | (0.235) |
| **Ln_Assets** | -0.305 | -0.350 | -0.360 | -0.359 |
|  | (0.251) | (0.255) | (0.265) | (0.255) |
| **LEV** | -0.267 | 0.152 | 0.295 | 0.032 |
|  | (0.803) | (0.856) | (0.908) | (0.864) |
| **SOE** | -0.127 | -0.173 | -0.272 | -0.107 |
|  | (0.360) | (0.357) | (0.367) | (0.358) |
| **Effect_Board** | 0.206 | 0.214 | 0.118 | 0.254 |
|  | (0.313) | (0.312) | (0.321) | (0.316) |
| **Total Income** | 0.288 | 0.232 | 0.243 | 0.243 |
|  | (0.229) | (0.230) | (0.243) | (0.230) |
| **OECD** | 0.518^*^ | 0.556^*^ | 1.069^***^ | 0.563^*^ |
|  | (0.283) | (0.284) | (0.356) | (0.301) |
| **Year Fixed Effects** | YES | YES | YES | YES |
| **Industry Fixed Effects** | YES | YES | YES | YES |
| ***N*** | 425 | 425 | 425 | 425 |
| ***R*^2^** | 0.0869 | 0.0929 | 0.1126 | 0.0978 |

t statistics in parentheses.

* p<0.1, ** p<0.05, *** p<0.01

**Comments:** Besides, the results are then difficult to interpret. The two institutional distances are very close together but give totally opposite results, which is difficult to understand and I am not convinced that it is not just a spurious result. When using CSR2, the variable becomes unsignificant.

I’m wondering about other variables that should be introduced in the model: valuation of the target firm (market to book), if it is a hostile transaction or growth of the target.

**Response:** Thank you very much for your detailed comments. Carefully following your suggestions, we have revised the measurements of the two institutional distances in order to lower correlation coefficients and reported the results shown above. What’s more, we also believe that if valuation of the target firm was introduced in the model would help us get interesting conclusions. However, limited by the availability of data, we cannot get any result as financial indicators of the target firm due to a large amount of missing data. Making full use of existing data, the OECD dummy variable measuring whether the target firm is from OECD countries is included in our model.

**Comments:** Endogeneity problems may arise. How did you deal with it? Would PSM or diff in diff be interesting in your case?

**Response:** Thank you very much for your kind and constructive comments.

Although we did some robustness tests including changing the econometric method and constructing new measurements of CSR, there are still potential endogeneity problems resulting from omitted variables. To ensure unbiased estimators, we use the Propensity Score Matching (PSM) method to run the regression (pp.22-24). Specifically, we divide the sample into two groups based on industry-mean CSR: a treatment group where companies have higher CSR scores than the mean in their respective industries and a comparison group with lower CSR. Then, we apply 1-to-1 nearest neighbor matching method within caliper to balance the observable individual characteristics between these two groups according to variables that influence the possibility of entering the treatment group. After matching, we estimate the model using the matched sample. The result in column (1) in Table 8 provides evidence consistent with the main conclusion, thus supporting our main analysis.

Moreover, we used an instrumental variable (IV) probit analysis. Drawing upon prior studies (El Ghoul et al. 2011; Harjoto and Jo 2015; Benlemlih and Bitar 2018; Ozdemir et al., 2021), we use industry-mean CSR as the instrumental variable for CSR. Industry-mean CSR is correlated to firm-level CSR as the intensity of CSR from peer companies in the same industry motivates companies to engage more in CSR activities, and it is not linked to cross-border M&A for a particular target (Ozdemir et al., 2021). The first stage regresses CSR on the instrumental variable (industry-mean CSR) with all other exogenous variables. Then, we regress cross-border M&A on the predicted CSR from the first stage. The results are reported in columns (2) and (3) in Table 8, respectively. The IV results are consistent with the basic findings, showing the robustness of our results.

**Table 8. PSM and IV Tests.**

|  | **(1)**  **PSM** | **(2)**  **The First Stage** | **(3)**  **The Second Stage** |
| --- | --- | --- | --- |
|  | Completion | CSR | Completion |
| **CSR** | 0.354^*^ |  | 0.837^***^ |
|  | (0.191) |  | (0.289) |
| **CSR_Mean(IV)** |  | 0.639^***^ |  |
|  |  | (0.094) |  |
| **Ln_Assets** | -0.350 | -0.004 | -0.056 |
|  | (0.255) | (0.039) | (0.091) |
| **LEV** | 0.152 | -0.891^***^ | 0.977^*^ |
|  | (0.856) | (0.277) | (0.507) |
| **SOE** | -0.173 | 0.197^***^ | -0.162 |
|  | (0.357) | (0.071) | (0.192) |
| **Effect_Board** | 0.214 | 0.082 | 0.127 |
|  | (0.312) | (0.079) | (0.173) |
| **TotalIncome** | 0.232 | 0.160^***^ | -0.113 |
|  | (0.230) | (0.052) | (0.104) |
| **OECD** | 0.556^*^ | -0.090 | 0.376^**^ |
|  | (0.284) | (0.066) | (0.156) |
| **Year Fixed Effect** | YES | YES | YES |
| **Industry Fixed Effect** | YES | YES | YES |
| ***N*** | 425 | 358 | 358 |
| ***R*^2^** | 0.0929 |  |  |

**Comments:** I’m not familiar with Hexun.com but some studies concerning China are using RKS, which could offer another interesting proxy for CSR.

**Response:** Thank you very much for your detailed comments. Mentioned in our original manuscript, we use CSR data from Hexun.com as it built the first vertical financial portal website in China and developed as an authorized financial securities information provider, as well as providing CSR score data for all listed companies according to CSR reports released on the official websites or in the annual reports issued by listed companies, thus providing us with accurate results. Following your suggestions, we also matched the CSR indicator from the RKS with our cross-border M&As data. However, the size of matched samples is just over 100 and there are many missing values, so we cannot run the regression using this small sample in order to get unbiased conclusion. In the future, we will continually investigate this study upon the availability of necessary data.

**Comments:** A few minor typos or awkward sentences:

(1.) “less gratifying”, “unavoidably”

(2.2) “There still a gap”

(3.2.2) “institutional diatance”

**Response:** Thank you very much for your detailed comments. Following your suggestions, we have revised these typos and awkward sentences with the help of professional copyeditor. Much more than this, we checked the whole manuscript supplemented with the latest revisions in carefulness and responsible attitude.

Thank you very much again for all your patience and insightful comments and suggestions. Hopefully, our response and revision could address your concerns. In any case, we believe that the quality of our paper has been improved with your kind help in this review process.

**Response to the Comments of Reviewer #2**

This paper investigates the impact of corporate social responsibility (CSR) on the completion of cross-border M&A with the moderating role of institutional distance over the years 2010-2016. The results suggest CSR has a positive role in the completion of the cross-border M&A of Chinese enterprises. Second, the formal institutional distance moderates the effect of CSR positively, whereas informal institutional distance moderates the effect negatively. In my view, this is a potentially interesting paper. However, I have the following concerns with this study.

**Response:** Thank you very much for your positive remarks on our paper. Very carefully following your guide, we have revised the paper, especially the part of methodology. Your comments have improved the quality of our paper significantly. Hopefully, our revisions could be satisfactory to you.

**Comments:** The paper should provide a table of the sample selection and exclusions. How is it distributed in time? Year-wise classifications between completed and non-completed cross-border M&A deals would add a clearer view of the sample. It should be better to have a distribution of target countries. I would also suggest you should consider providing descriptive statistics of all variables used in this study between the completed and non-completed M&A deals and test between these two groups using parametric and non-parametric tests to see if there are any significant differences between the above two groups.

**Response:** Thank you very much for your insightful comments. Following your suggestions, we present year-wise classifications between completed and non-completed cross-border M&A deals, a distribution of target countries between these two groups, summary statistics of all variables classified according to the sample mean and median of the CSR scores as well as testing the significance of differences. These results are provided in Table 2, Table 3 and Table 4 respectively (pp.13-15). As shown in Table 3, firms completing cross-border M&A deals have higher CSR scores than firms with failure of overseas M&A, suggesting that firm with higher CSR are more possible to succeed in cross-border M&A deals.

**Table 2. Sample Distribution by Year.**

| **Year** | **Completion=0** | **Completion=1** | **Total** |
| --- | --- | --- | --- |
| **2010** | 18 | 14 | 32 |
| **2011** | 16 | 19 | 35 |
| **2012** | 28 | 18 | 46 |
| **2013** | 27 | 28 | 55 |
| **2014** | 43 | 44 | 87 |
| **2015** | 75 | 57 | 132 |
| **2016** | 28 | 34 | 62 |
| **Total** | 235 | 214 | 449 |

**Table 3. Sample Distribution by Target Countries.**

| **Target Country** | **Completion=0** | **Completion=1** | **Total** |
| --- | --- | --- | --- |
| **America** | 54 | 57 | 111 |
| **Argentina** | 2 | 1 | 3 |
| **Australia** | 22 | 20 | 42 |
| **Austria** | 1 | 3 | 4 |
| **Brazil** | 5 | 4 | 9 |
| **Canada** | 16 | 16 | 32 |
| **Denmark** | 3 | 3 | 6 |
| **Finland** | 3 | 1 | 4 |
| **France** | 9 | 7 | 16 |
| **German** | 28 | 19 | 47 |
| **India** | 3 | 1 | 4 |
| **Indonesia** | 4 | 1 | 5 |
| **Italy** | 6 | 14 | 20 |
| **Japan** | 6 | 9 | 15 |
| **Malaysia** | 3 | 3 | 6 |
| **Netherlands** | 8 | 4 | 12 |
| **Newsela** | 1 | 2 | 3 |
| **Norway** | 2 | 1 | 3 |
| **Pakistan** | 5 | 0 | 5 |
| **Poland** | 2 | 1 | 3 |
| **Singapore** | 13 | 4 | 17 |
| **Spain** | 1 | 5 | 6 |
| **Switzerland** | 3 | 3 | 6 |
| **Thailand** | 6 | 2 | 8 |
| **Vietnam** | 2 | 1 | 3 |
| **Others** | 30 | 35 | 65 |
| **Total** | 235 | 214 | 449 |

**Table 4. Summary Statistics by Completion.**

| **Variables** | **Completion=0** | **Mean0** | **Completion=1** | **Mean1** | **Test of Difference** |
| --- | --- | --- | --- | --- | --- |
| **CSR** | 235 | 3.154 | 214 | 3.325 | -0.170^**^ |
| **Formal Institutional Distance** | 235 | 1.118 | 214 | 1.053 | 0.065 |
| **Informal Institutional Distance** | 235 | 1.093 | 214 | 1.107 | -0.014 |
| **Ln_Assets** | 235 | 22.8 | 214 | 22.9 | -0.1 |
| **LEV** | 235 | 0.451 | 214 | 0.461 | -0.01 |
| **SOE** | 235 | 0.294 | 214 | 0.304 | -0.01 |
| **Effect Board** | 235 | 0.787 | 214 | 0.804 | -0.017 |
| **Total Income** | 235 | 21.98 | 214 | 22.1 | -0.117 |
| **OECD** | 235 | 0.719 | 214 | 0.808 | -0.089^**^ |

**Comments:** The biggest concern in the analysis is correlated omitted variables. There could be unobserved factors that are correlated with CSR, institutional distance and control variables. This raises concerns regarding omitted variable bias in your probit regression results. My suggestion is to run an instrumental variable (IV) probit model to address the potential endogeneity concern.

**Response:** Thank you very much for your constructive suggestions.

To address concerns of potential endogeneity problem arising from omitted variables, we have done the instrumental variable (IV) probit analysis as you suggested (pp. 23-24). Drawing upon prior studies (El Ghoul et al. 2011; Harjoto and Jo 2015; Benlemlih and Bitar 2018; Ozdemir et al., 2021), we use industry-mean CSR as the instrumental variable for CSR. Industry-mean CSR is correlated to firm-level CSR as the intensity of CSR from peer companies in the same industry motivates companies to engage more in CSR activities, and it is not linked to cross-border M&A for a particular target (Ozdemir et al., 2021). The first stage regresses CSR on the instrumental variable (industry-mean CSR) with all other exogenous variables. Then, we regress cross-border M&A on the predicted CSR from the first stage. The results are reported in columns (2) and (3) in Table 8, respectively. The IV results are consistent with the basic findings, showing the robustness of our results.

Moreover, we use Propensity Score Matching (PSM) method to run the regression following your suggestions (pp.22-24). Specifically, we divide the sample into two groups based on industry-mean CSR: a treatment group where companies have higher CSR scores than the mean in their respective industries and a comparison group with lower CSR. Then, we apply 1-to-1 nearest neighbor matching method within caliper to balance the observable individual characteristics between these two groups according to variables that influence the possibility of entering the treatment group. After matching, we estimate the model using the matched sample. The result in column (1) in Table 8 provides evidence consistent with the main conclusion, thus supporting our main analysis.

**Table 8. PSM and IV Tests.**

|  | **(1)**  **PSM** | **(2)**  **The First Stage** | **(3)**  **The Second Stage** |
| --- | --- | --- | --- |
|  | Completion | CSR | Completion |
| **CSR** | 0.354^*^ |  | 0.837^***^ |
|  | (0.191) |  | (0.289) |
| **CSR_Mean(IV)** |  | 0.639^***^ |  |
|  |  | (0.094) |  |
| **Ln_Assets** | -0.350 | -0.004 | -0.056 |
|  | (0.255) | (0.039) | (0.091) |
| **LEV** | 0.152 | -0.891^***^ | 0.977^*^ |
|  | (0.856) | (0.277) | (0.507) |
| **SOE** | -0.173 | 0.197^***^ | -0.162 |
|  | (0.357) | (0.071) | (0.192) |
| **Effect Board** | 0.214 | 0.082 | 0.127 |
|  | (0.312) | (0.079) | (0.173) |
| **Total Income** | 0.232 | 0.160^***^ | -0.113 |
|  | (0.230) | (0.052) | (0.104) |
| **OECD** | 0.556^*^ | -0.090 | 0.376^**^ |
|  | (0.284) | (0.066) | (0.156) |
| **Year Fixed Effect** | YES | YES | YES |
| **Industry Fixed Effect** | YES | YES | YES |
| ***N*** | 425 | 358 | 358 |
| ***R*^2^** | 0.0929 |  |  |

**Comments:** This study examines the impact of CSR on the completion of cross-border M&A using a logit model. The dependent variable (Completion) is a binary variable that whether the cross-border M&A was completed or not. I would suggest that you should calculate short-run abnormal returns and long-run performance around the cross-border M&A deals and test the moderating effect of CSR and institutional distance on the calculated performance.

**Response:** Thank you very much for your constructive comments. Your comments provide us another insightful implication to move our research forward in the future.

First, the completion of M&A has been attracting the academic attentions, especially involving emerging market (e.g., Zhou, Xie, and Wang, 2016; Dikova, Sahib, and Witteloostuijn, 2010). Additionally, there is a high percentage collapse before completion (Zhou, Xie, and Wang, 2016). Therefore, it is meaningful to discuss how to enhance the success of M&A.

Second, we definitely believe that it is a very interesting topic to discuss the influence of CSR and institutional distance on the abnormal returns caused by M&A deals. However, we suppose this is another topic that is different from the current topic exploring the influence of CSR and institutional distance on the completion of M&A. Furthermore, as you suggested, it seems that CSR may has an indirect effect (e.g., the moderating effect) on the abnormal returns caused by M&A, as the direct link between CSR and the abnormal returns caused by M&A is not very clear. Thus, we need to develop very strong arguments different with current study to answer why and how CSR affects the abnormal returns closely related to the event of M&A. After careful consideration, we suppose that it is better to regard it as quite another research rather than integrating it into the current topic. We appreciate your inspiring comments leading us to conduct potential research in the future.

Thank you very much again for all your patience and insightful comments and suggestions. Hopefully, our response and revision could address your concerns. In any case, we believe that the quality of our paper has been improved with your kind help in this review process.
