# Supplementary material for: Corporate social responsibility and cross-border M&A: The moderating effect of institutional distance
Source: PLoS One. 2022 Jan 27;17(1):e0262343. doi: 10.1371/journal.pone.0262343 (PMC8794142; doi:10.1371/journal.pone.0262343)
Supplement: Attachment — Submitted filename: Response to reviewers.docx [file pone.0262343.s002.docx]

PONE-D-21-18638

**Response to the Comments of Academic Editor**

**Comments:** Thank you for submitting your manuscript to PLOS ONE. After careful consideration, we feel that it has merit but does not fully meet PLOS ONE’s publication criteria as it currently stands. Therefore, we invite you to submit a revised version of the manuscript that addresses the points raised during the review process.

The paper still requires further revisions with reference to empirical design, discussion, as well as paper contribution.

**Response:** Thank you very much for your positive remarks on our manuscript. We very much appreciate you allowing us to revise and improve the paper again. Following the comments of you and Reviewer 1, we have carefully revised the empirical design, clearly clarify our contribution and discussion. We hope you and the reviewer will find our response and revised manuscript satisfactory.

**Response to the Comments of Reviewer #1**

**Comments:** Dear authors,

Thanks for your work on your research. I think the Chinese context is now well explained, the hypothesis and the paper more clear overall.

I, however, still have a few comments:

**Response:** Thank you very much for your positive remarks on our paper. Following your suggestions, we have carefully revised the paper again. We are very sure that your remarks have improved the quality of this paper greatly. Specifically, your constructive suggestions have helped us to resolve the major concerns from measures and model specifications and make the contributions of our paper much clearer and accurate.

**Comments:** Measures

The way you measure formal and informal distance is not clear. For the FID, you use absolute values whereas, most of the time, Euclidean distance is preferred (see Wanli Li, Chaohui Wang, Qizhe Ren, Ding Zhao, 2020, Institutional distance and cross-border M&A performance: A dynamic perspective, Journal of International Financial Markets, Institutions and Money, Volume 66). You mention Fi but they are not really defined: is it factors from the WBI? Concerning informal distance, I do not understand why you only use one dimension of Hofstede. Keig and al [46] use four of the dimensions, not one.

**Response:** Thank you very much for your kind and constructive suggestions. Carefully following your suggestions, we have revised the measurements and calculations of institutional distance. Now, we believe that the measurements are more reliable and accurate.

In reference to Li et al. (2020) as you suggested, we have re-calculated both formal and informal institutional distance by using the Euclidean distance.

In this study, we use the Worldwide Governance Indicators (WGI) to measure formal institutions. The WGI project measures the development of a country’s institutions by using six indicators: (1) Voice and Accountability; (2) Political Stability and Absence of Violence; (3) Government Effectiveness; (4) Regulatory Quality; (5) Rule of Law; and (6) Control of Corruption. Specifically, ${FID}_{j}$ represents the formal institutional distance between the host country j and the home country, $I_{nj}$ is the score of dimension n for the host country in year t, $I_{nc}$ denotes the score of dimension n for the home country in the year t, and $V_{n}$ denotes the variance of dimension n in the same year.

$${FID}_{j}=\sqrt{\sum_{n}^{6} \frac{{(I_{nj}-I_{nc})}^{2}}{V_{n}}}$$

In the prior version, we argue that uncertainty avoidance, one of Hofstede’s cultural dimensions, is more closely related with the perception and acceptance of MNCs’ CSR practices from stakeholders in host country, thereby selecting it to measure informal institutions.

In the new revision, following your suggestions, we measured informal institutions by using the whole six dimensions of Geert Hofstede's national culture: (1) Individualism; (2) Power Distance; (3) Masculinity; (4) Uncertainty Avoidance; (5) Long-term Orientation; and (6) Indulgence. And also, we re-calculated the informal institutional distance by using the Euclidean distance.

$${IID}_{j}=\sqrt{\sum_{m}^{6} \frac{{(I_{mj}-I_{mc})}^{2}}{V_{m}}}$$

**Comments:** Specification

I’m still confused by your specification since there are important missing variables (like valuation, cash, firm growth if there is a runup, the age of the firm…) and in the same time, most of the variables (as size) do not display a significant relationship. How could we convince us that the model is correctly specified?

**Response:** Thank you very much for your detailed and constructive comments about the model specification. We very much agree with you that the possible omission of some important variables may influence the accuracy of our regression estimations. To resolve the concerns about the misspecification of model, especially the omission of some important variables, we made a major revision on the model specification.

First, besides of year, industry, and region fixed effect, we added firm fixed effect as well, so that we could rigorously control for all other non-observational firm-level variables in the model, thereby ensuring the estimation accuracy.

Second, at the merging and acquiring enterprise level, this paper controls for the following variables: (1) Firm age (Ln_Age) is the natural logarithm of the number of years since the merging and acquiring company started operating. (2) Firm size (Ln_Assets) is the natural logarithm of the total assets of the merging and acquiring company at the end of the year. (3) Cash flow (Cash_ratio) is the ratio of the sum of cash and cash equivalent to current liabilities, which reflects the operation of enterprise. (4) Working capital ratio (Flow_ratio) is the ratio of current assets to current liabilities measuring the risk of operation, which is vital for cross-border M&A. (5) Asset_liability_ratio is the ratio of total liabilities to total assets, which is a measurement of financial leverage. (6) Equity ownership (SOE), which is a binary variable equal to 1 if the acquiring company is a state-owned enterprise, and 0 otherwise. State ownership ties are likely to influence the regulated resources and policy treatment that a firm can acquire from the government, which may affect the competition of an M&A event. (7) Competition is measured by the number of competing buyers for overseas M&A.

Further, the IV estimation method could also reduce our concern about model misspecification caused by the missing variables.

Table 1 reports the estimated Logit model results based on the revised model specification. Model (1) is the baseline regression. Model (2) reports the regression results with control variables. In Model (2), the regression coefficients of controls such as Ln_Assets, Flow_ratio and Asset_liability_ratio are significantly positive, denoting that the good financial performances of the acquiring enterprises could motivate overseas M&A. The regression coefficient of CSR is significantly positive, indicating that CSR has a significant positive relationship with Chinese enterprises’ cross-border M&A. Model (3) and model (4) show the moderating effects of formal institutional distance and informal institutional distance respectively after controlling all fixed effects and variables.

Specifically, upon carefully revised the measurement and model specification, we finally found a positive moderating effect of informal institutional distance, in opposite to our prior findings. To re-confirm the consistence of the moderating effect, we conducted a series of robustness check for it and still found the same result. Therefore, we very much believe that the significant improvement of the measurement and model specification lead to the “opposite” but “appropriate” regression estimation.

We appreciate very much for your great help! Your insightful suggestions help us to correct the model specification and variable measurements.

**Table 1 Basic Results and Moderating Effects**

|  | **(1)** | **(2)** | **(3)** | **(4)** |
| --- | --- | --- | --- | --- |
|  | **Completion** | **Completion** | **Completion** | **Completion** |
| **CSR** | 1.221^***^ | 1.299^**^ | 0.734 | -4.721 |
|  | (0.467) | (0.655) | (1.238) | (2.876) |
| **Formal Institutional Distance** |  |  | 0.453 |  |
|  |  |  | (0.449) |  |
| **CSR* Formal Institution Distance** |  |  | 1.273^*^ |  |
|  |  |  | (0.705) |  |
| **Informal Institutional Distance** |  |  |  | 0.433 |
|  |  |  |  | (0.482) |
| **CSR* Informal Institution Distance** |  |  |  | 2.902^*^ |
|  |  |  |  | (1.679) |
| **Ln_Age** |  | 9.858 | 16.094 | 15.585 |
|  |  | (9.406) | (15.202) | (15.698) |
| **Ln_Assets** |  | 2.694^*^ | 5.473^**^ | 3.763 |
|  |  | (1.422) | (2.452) | (3.910) |
| **Cash_ratio** |  | -1.209 | -1.883 | 1.228 |
|  |  | (1.149) | (2.432) | (4.190) |
| **Flow_ratio** |  | 1.220^*^ | 2.288 | -0.305 |
|  |  | (0.688) | (1.623) | (1.339) |
| **Asset_liability_ratio** |  | 15.570^***^ | 13.052^*^ | -0.668 |
|  |  | (5.192) | (7.823) | (16.162) |
| **SOE** |  | -0.014 | 1.524 | 0.960 |
|  |  | (2.018) | (2.503) | (6.546) |
| **Competition** |  | 1.267 | 1.229 | 1.080 |
|  |  | (1.663) | (2.823) | (2.796) |
| **Year Fixed Effects**  **Firm Fixed Effects** | YES  YES | YES  YES | YES  YES | YES  YES |
| **Industry Fixed Effects**  **Province Fixed Effects** | YES  YES | YES  YES | YES  YES | YES  YES |
| ***R*^2^** | 0.1914 | 0.2577 | 0.2836 | 0.3192 |

**Comments:** Robustness

Concerning the robustness tests, they are performed on H1, but not on what is the most interesting in the paper, H2 and H3. This is an important issue for the robustness of your findings. I’m also not really convinced by your explanation of the counterintuitive results of H3. This reference may help you: Javier Aguilera-Caracuel, Nuria Esther Hurtado-Torres, Juan Alberto Aragón-Correa, Alan M. Rugman, 2013, Differentiated effects of formal and informal institutional distance between countries on the environmental performance of multinational enterprises, Journal of Business Research, Volume 66, Issue 12.

**Response:** Thank you very much for your insightful comments. Following your suggestions, we further conducted the robustness tests for the moderating effects through changing the measurement and calculation of institutional distance, and we still found the consistent results. For example, we used the absolute value to measure institutional distance, as displayed in Model (1)-(2) in Table 2. And also, we changed the factors to measure institutional distances. For formal institutional distance, the four factors of political stability, absence of violence, government effectiveness, and regulatory quality were selected. For informal institutional distance, the four indicators of power distance, individualism-collectivism, uncertainty avoidance, and masculinity-femininity were selected.

Thank you very much again for your insightful comments, especially your persistent focus on the “counterintuitive” moderating effect of informal institutional distance and your great help on model specification and variable measurement.

**Table 2** **Robustness Tests of Moderating Effects**

|  | **(1)**  **Completion** | **(2)**  **Completion** | **(3)**  **Completion** | **(4)**  **Completion** |
| --- | --- | --- | --- | --- |
| **CSR** | 1.066 | -14.770^**^ | 0.665 | -5.262^**^ |
|  | (1.309) | (6.536) | (1.241) | (2.346) |
| **Absolute Formal Distance** | 1.544 |  |  |  |
|  | (1.172) |  |  |  |
| **CSR* Absolute Formal Distance** | 3.166^**^ |  |  |  |
|  | (1.410) |  |  |  |
| **Absolute Informal Distance** |  | 9.848^*^ |  |  |
|  |  | (5.326) |  |  |
| **CSR* Absolute Informal Distance** |  | 26.302^*^ |  |  |
|  |  | (14.356) |  |  |
| **4-dimension Formal Distance** |  |  | 0.440 |  |
|  |  |  | (0.510) |  |
| **CSR*4-dimension Formal Distance** |  |  | 1.787^*^ |  |
|  |  |  | (0.959) |  |
| **4-dimension Informal Distance** |  |  |  | 1.654^*^ |
|  |  |  |  | (0.886) |
| **CSR*4-dimension Informal Distance** |  |  |  | 5.861^*^ |
|  |  |  |  | (3.525) |
| **Ln_Age** | 18.302 | 13.384 | 14.209 | 51.693^**^ |
|  | (15.595) | (15.625) | (15.180) | (24.521) |
| **Ln_Assets** | 5.834^**^ | 2.183 | 5.338^**^ | 6.310 |
|  | (2.505) | (4.206) | (2.472) | (4.302) |
| **Cash_ratio** | -1.748 | 15.743 | -1.877 | 1.906 |
|  | (2.406) | (11.658) | (2.420) | (5.350) |
| **Flow_ratio** | 2.164 | -5.054 | 2.278 | 0.180 |
|  | (1.623) | (3.842) | (1.625) | (1.554) |
| **Asset_liability_ratio** | 10.654 | 16.006 | 13.089^*^ | 14.346 |
|  | (8.163) | (27.742) | (7.894) | (21.268) |
| **SOE** | 1.420 | 20.780 | 1.592 | 4.881 |
|  | (2.625) | (21.988) | (2.537) | (7.900) |
| **Competition** | 1.267 | 0.693 | 1.215 | 0.883 |
|  | (2.865) | (2.317) | (2.807) | (2.631) |
| **Year Fixed Effects**  **Firm Fixed Effects**  **Industry Fixed Effects**  **Province Fixed Effects** | YES  YES  YES  YES | YES  YES  YES  YES | YES  YES  YES  YES | YES  YES  YES  YES |
| ***R*^2^** | 0.2902 | 0.4334 | 0.2824 | 0.3593 |

**Comments:** Contribution

Besides, you should explain your contribution compared to: Yang, K., & Boasson, V. (2021). Cross-border acquisitions and institutional distance: does country connectedness matter? International Journal of Business and Globalisation, 28(3), 332-348.

I hope these comments will improve your work.

**Response:** Thank you very much for your kind and constructive comments. Definitely, your guide has greatly helped us to improve the quality of our paper, make our results more reliable, and refine the theoretical contributions. We appreciate your suggestions and help to our manuscript greatly.

Specifically, we carefully read the reference of Yang and Boasson (2021) you suggested and reconsider our “uniqueness” from Yang and Boasson (2021), and then clarify the theoretical contributions again.

First, the research focuses of our study significantly differ from Yang and Boasson (2021). Yang and Boasson (2021) focuses on the main effect of institutional distance on cross-border M&As, especially the interaction of formal and informal institutional distance, whilst this study focuses on the vital role of corporate social responsibility in cross-border M&As, especially its positive effect on overcoming the barriers of institutional distance.

Second, this study contributes to the literature relevant with the influence of institutional distance on M&A (e.g., Yang and Boasson, 2021) through investigating the positive role of CSR on overcoming the negative effect of institutional distances. That is, different from the prior studies just focusing on the negative influence of institutional distances, this study further explores how firms actively do to weaken or remove these negative influences caused by a high level of institutional distance.

Thank you very much again! We appreciate for your great help to correct our model and improve the quality of our paper. Hopefully, our revisions could make you satisfactory.

References:

1. Li WL, Wang CH, Ren QZ, Zhao D. Institutional distance and cross-border M&A performance: A dynamic perspective. Journal of International Financial Markets, Institutions and Money. 2020;66. doi: [10.1016/j.intfin.2020.101207](https://schlr.cnki.net/Detail/doi/SJESLAST/SJES5DF728A941189F56D500C77EC36CD807).
2. Kisgen DJ, Qian J, Song W. Are fairness opinions fair? The case of mergers and acquisitions. J Financ Econ. 2009;91(2):179-207. doi: [10.1016/j.jfineco.2008.03.001](https://doi.org/10.1016/j.jfineco.2008.03.001).
3. Chen Y, Huang Y, Chen C. Financing constraints, ownership control, and cross-border M&As: evidence from nine East Asian economies. Corp Gov Int Rev. 2009;17: 665-680. doi: [10.1111/j.1467-8683.2009.00770.x](https://doi.org/10.1111/j.1467-8683.2009.00770.x).
4. Xia J, Ma X, Lu JW, Yiu DW. Outward foreign direct investment by emerging market firms: a resource dependence logic. Strateg Manag J. 2014;35(9):1343-1363. doi: [10.1002/smj.2157](https://doi.org/10.1002/smj.2157).
5. Tang Q, Gu FF, Xie E, Wu Z. Exploratory and exploitative OFDI from emerging markets: impacts on firm performance. Int Bus Rev. 2020; 29:101661. doi: [10.1016/j.ibusrev.2019.101661](https://doi.org/10.1016/j.ibusrev.2019.101661).
6. Keig DL, Brouthers LE, Marshall VB. The impact of formal and informal institutional distances on MNE corporate social performance. Int Bus Rev. 2019;28:85-92. doi: [10.1016/j.ibusrev.2019.05.004](https://doi.org/10.1016/j.ibusrev.2019.05.004).
