# Supplementary material for: Corporate social responsibility and cross-border M&A: The moderating effect of institutional distance
Source: PLoS One. 2022 Jan 27;17(1):e0262343. doi: 10.1371/journal.pone.0262343 (PMC8794142; doi:10.1371/journal.pone.0262343)
Supplement: Attachment — Submitted filename: Response to Reviewers.docx [file pone.0262343.s003.docx]

PONE-D-21-18638

**Response:** Thank you very much for your positive remarks on our manuscript. We arr thankful for allowing us to revise and improve the paper again. Following the comments of you and Reviewer 3, we have carefully revised the expression of hypothesis, clarified our empirical model and corrected typos. We hope you and the reviewer will find our responses and revision work satisfactory.

**Response to the Comments of Reviewer #3**

**Comments:** The authors have followed the previous comments from the editor and reviewers to make revisions in accordance.

**Response:** We very much appreciate your recognition on our revised work. Following the comments from you, we have carefully revised the expression of hypothesis, clarified our empirical model and corrected typos. We are very sure that your comments have improved the quality of this paper greatly.

**Comments:** The statements in hypotheses 1 to 3 are not consistent with the usual academic writing. In a hypothesis, there will not be the term ‘significantly’. Significance (or insignificance) is the result from statistical tests. Therefore, Hypothesis 1 may be like “CSR has a positive effect on cross-border M&A.” Usually there will not be a sentence of “That is,…” in a research hypothesis. Research hypotheses 2 and 3 are also strange because they are not expressed in complete sentences. They may be revised as “M positively (or negatively) moderates the effect of X on Y.

**Response:** Thank you very much for your kind and detailed suggestions. Following your suggestions, we have revised the statements in hypotheses 1 to 3.

**Comments:** However, the dataset are during 2010-2016. Tables 6-7 are results from fixed-effects panel regressions. Since each M&A is a one-shot event, maybe it is fine to use fixed-effects regression instead of random-effects data regression. The authors need to justify why a fixed-effects Logit regression is appropriate to use for the data analysis. If possible, some statistical tests should be provided to justify the use of fixed-effects regressions.

**Response:** Thank you very much for your kind and constructive suggestions. We totally agree with you that the M&A is one-shot event, and our dataset is not a panel data although it is distributed from 2010 to 2017. Thus, the statement about the model we need to clarify first is that our empirical model is not a fixed-effect panel regression model but a pooled cross section model. We are very sorry about the inappropriate statements using the word of “fixed effect” in Table 6, Table 7, and others. We have revised all the statements in the manuscript and clarified that we just added the relevant dummies but not used the fixed-effect model.

Specifically, considering that some unobserved factors related with time, region, and industry may influence cross-border M&As, we generated firm, year, industry, and province dummies and added them into the model, thereby enhancing the model rigorousness and ensuring the estimation accuracy. This specification can also be seen in prior studies (e.g., Li et al., 2020; Gomes, 2019).

Thank you very much again for your careful and professional suggestions.

**Comments:** The current conclusion now can be directly supported from the empirical findings in this paper. The authors’ efforts in revising this paper should be positively affirmed.

**Response:** Thank you very much for your positive remarks on our efforts. Your constructive suggestions greatly help us improve the quality of this paper.

**Comments:** There are still some typos and format problems in the revised manuscript which need to be carefully corrected. For instance, in the Figure, H1, H2, and H3 cover parts of the arrows (paths). On page 38, it shows “42. 42.”

**Response:** Thank you very much for your kind and constructive suggestions. Following your advice, we went through the whole paper very carefully and corrected the typos and problems.

Thank you very much again! We greatly appreciate your help and suggestions. Hopefully, you will find our revisions satisfactory.

References:

[1] Li WL, Wang CH, Ren QZ, Zhao D. Institutional distance and cross-border M&A performance: A dynamic perspective. Journal of International Financial Markets, Institutions and Money. 2020;66. doi: 10.1016/j.intfin.2020.101207.

[2] Gomes M. Does CSR influence M&A target choices? Finance Research Letters. 2019; 30: 153–159. DOI:10.1016/J.FRL.2018.09.011.
